# Validation of a viable delirium detection test performed by nurses and physicians during routine patient care

**DOI:** 10.1186/s12877-024-04884-8

**Published:** 2024-03-28

**Authors:** Rashad Soboh, Sharon Gino-Moor, Nizar Jiris, Shira Ginsberg, Ron Oliven

**Affiliations:** 1 Fliman Geriatric Hospital, Haifa, Israel; 2 Department of Medicine C, Bnai Zion Medical Center, 47 Golomb str, 3339419 Haifa, Israel; 3 Rappaport School of Medicine, Technion Institute of Technology, Haifa, Israel

**Keywords:** Delirium, Diagnosis, Validity, 4AT, Non-inferiority

## Abstract

**Background:**

Delirium is a frequent mental impairment in geriatric patients hospitalized in acute care facilities. It carries a high risk of complications and is often the first symptom of acute illness. It is clearly important to identify the development of delirium at an early stage, and several short and effective diagnostic tests have been developed and validated for this purpose. Despite this, patients on hospital wards are seldom monitored for signs of emergent delirium, suggesting that compliance with guidelines would be improved by introducing a simpler and more user-friendly test.

**Methods:**

We recently implemented a simple delirium assessment tool, called RMA that can be introduced into the daily routine of ward staff without significantly adding to their workload. The nurses noted their impression of the patient’s cognitive state in the electronic medical record, and during the morning round the ward physician administered a short attention test to any patients suspected of new cognitive impairment. In this study, we compared RMA test against the widely used and well validated 4AT.

**Results:**

RMA performed daily by the ward staff was found to be non-inferior to 4AT performed by an experienced rater. Compared to 4AT, R&M had a sensitivity of 93.9% and a specificity of 98.3%. An Altman-Bland plot indicated that both tests can be used interchangeably.

**Conclusions:**

The RMA test is reliable, easy to administer, likely to boost compliance with guidelines, and is expected to raise awareness of delirium among the nurses and physicians directly involved in the diagnostic process.

## Introduction

Delirium is characterized by an altered mental status and/or a confusional state that develops acutely and tends to have a fluctuating course [1]. Although delirium has been known since ancient times, the condition is often not diagnosed, documented, evaluated, and managed [2]. Delirium is most common among hospitalized geriatric patients, and more than a quarter of all patients in internal medicine wards aged 70 and over suffer from delirium [3–6].

Early detection of delirium may be critical as mental deterioration in the elderly can be the first sign of diseases and disorders that precipitate delirium [7–10].

Previous studies have shown that delirium is only partially identified by the treating staff [4–6, 11, 12]. The main reason for this shortcoming is probably insufficient awareness of the importance of early diagnosis, although lack of time may be a contributing factor. There are a number of short tests that ward staff can learn and administer in a few minutes [13–20]. Of these, the CAM and 4AT have been most thoroughly validated [13–15]. Nevertheless, we [21] and others [22, 23] found that in the real world, both CAM and 4AT usually require more than a few minutes to provide reliable results, and residents are often reluctant to perform daily mental assessments in each elderly patient.

Recently, Voyer et al. developed a delirium screening tool (RADAR) that can be administered by nurses as part of their daily routine [22–24]. RADAR is very time-efficient, and its sensitivity ranged from 70 to 100% in different clinical settings when compared to CAM [22–24]. RADAR provides only a partial evaluation of delirium, and was proposed by the authors as a screening test (“6th vital sign”). As RADAR is not specific for delirium (in previous studies a specificity of 67–83% was reported), its specificity needs to be improved for it to be useful in clinical practice. Nevertheless, the notion of nurse-administered pre-screening is appealing: if the nurse’s evaluation is sufficiently sensitive, RADAR may reduce the number of patients requiring further evaluation.

To test this hypothesis, RADAR was included in our department’s electronic medical records. An abnormal RADAR activated an alert flag in the medical field, and the resident then had to confirm the presence of delirium during the morning round. For this purpose we chose the **M**onths **O**f the **Y**ear **B**ackwards (MOYB) test [25], a short assessment included also in other brief delirium tests [14, 18, 19]. Adding the MOYB test to the delirium diagnosis protocol had several goals: corroboration the observational finding of the nurses; improving the specificity of the test; confirming the presence of inadequate attention/concentration, considered essential for diagnosing delirium, also by the delirium definition of DSM-5; and not less important in our opinion– involving the residents in the delirium diagnosis procedure, thereby increasing their awareness to this often missed complication and their adherence to the diagnostic and therapeutic guidelines that should be followed in patients who develop delirium.

Both RADAR and MOYB are tests that, when positive, suggest the presence of a cognitive impairment (CI). In order to diagnose delirium, it must be shown that the identified CI is acute, with a new onset, and not chronic, such as dementia. Confirming a recent alteration in CI found is the final step in patients with positive RADAR and MOYB. We named RADAR combined with MOYB and assessment of the recency of CI, if found, the “RMA” test, with A standing for acute or alteration. Both a positive RADAR + abnormal MOYB + new onset were required for the RMA test to be abnormal, thereby diagnosing delirium.

The purpose of this study was to evaluate the validity and reliability of the new RMA test by comparing it to the widely used, fully validated and reliable 4AT. The 4AT was chosen as comparator due to its close similarity to the RMA test.

## Methods

Before implementing the RMA in routine ward rounds, the head of the hospital’s geriatric unit (RO) gave the staff (nurses and residents) comprehensive instructions and training in the administration of their respective tests. An RMA field was added to the electronic medical records. At the start of RADAR implementation, a geriatric nurse conducted spot scans of the nurses’ records to verify they were filled out as required. Six months after RMA had been implemented in the department’s routine practice, the 4AT (**A**ttention, **A**bbreviated mental test, **A**lertness, **A**cute onset **T**est) was administered by a resident in geriatrics with previous experience with this test (RS), as part of the implementation process, to ratify the reliability of RMA. The results of the 4AT were also entered into the patients’ medical records. The current manuscript reports the results of a retrospective analysis of the data extracted from the patients’ records, performed after a sufficient number of patients underwent both tests. The requirement for informed consent was waived by the Ethics Committee of Bnai Zion Medical Center because of the retrospective nature of the study.

### Subjects

Patients aged over 70 years, hospitalized in the internal medicine wards in the Bnai-Zion Medical Center, a municipal hospital affiliated with the faculty of medicine, were included in this study. Patients with advanced dementia and disability (based on referral letters) referred from nursing homes, terminally ill patients and patients unable to cooperate due to language barriers were excluded. The study was approved by the Human Investigations Review Board of Bnai Zion Medical Center, Haifa, Israel.

### Delirium tests

The RMA test was administered every day to each patient by the ward staff during the morning hours. Unlike RMA, the 4AT was performed randomly 1–3 times a week on weekdays, at noon, within 4 h from RMA. The 4AT was only administered once in a given patient.

The RMA test was performed in 3 stages: first, the RADAR **(R**ecognizing **A**cute **D**elirium **A**s part of your **R**outine) test was administered to all elderly patients (age > 70 yrs) by the nurse based on her observation of the patient while dispensing the morning drugs, as previously described [22, 23]. In the electronic medical records, nurses answered “yes” or “no” to 3 questions: (1) Was the patient unusually sleepy? (2) Did the patient have difficulty following instructions? (3) Were the patient’s movements unusually slow? If the answer was “yes” to any one of the questions, the RADAR test was considered positive and a red flag appeared in the resident’s field of the patient’s electronic medical records, indicating possible delirium.

Secondly, every patient whose records indicated a positive RADAR test was evaluated by their treating physicians using the MOYB during their routine morning rounds to confirm the presence of cognitive impairment. MOYB was performed as described in 4AT (Table 1), but for RMA it was scored only as positive or negative, i.e., MOYB was considered positive (pathological) if the patient was unable to say 7 or more months of the year in backward order, starting with December, or MOYB was untestable. An abnormal MOYB confirmed the presence of an attention/concentration deficit, and was used, together with the nurse’s assessment (i.e., positive RADAR), to diagnose the presence of cognitive disturbance.

**Table 1:** 4AT parameters and scoring (https://www.the4at.com)

4AT parameters	evaluation	scoring
**A**lertness	drowsiness/sleepiness	normal or mild brief sleepiness 0 clearly abnormal 4
**A**MT4	age, date of birth, place, current year	no mistake 0 1 mistake 1 2 or more mistakes 2
**A**ttention (MOYB)	ability to say the months of the year in backward order, starting with December	> 6 months 0 < 7 months 1 Untestable 2
**A**cute change in cognition	acute or fluctuating change in mental function	no0 yes4

AMT4– Abbreviated Mental Test; MOYB– Months Of the Year Backwards Test

Scoring key: acute (including fluctuating) change in cognition was considered mandatory for the diagnosis of delirium. Accordingly, a score > 4 in the presence of a new change in cognition was defined as delirium

Finally, CI, based on positive RADAR + positive MOYB, could be defined as delirium only if it was reported to be new or fluctuating relative to the earlier patient’s mental state. This information was provided by the patient’s relatives, friends or caregivers on the day of admission, and later on it was based on assessments and acquaintance with the patient from previous days. If the same level of CI was reported to be pre-existing, it was labeled as “chronic stable” CI (CCI), usually due to dementia. Delirium superimposed on dementia was counted as delirium.

Performing 4AT was started after a sufficient test-run of RMA, and used to validate the reliability of the new test. The 4AT (Table 1) was performed as previously described [14]. Only records that included all required RMA data obtained within the predetermined 4 h time-span were used. The ward staff’s RMA scores were not accessible to the physician who performed the 4AT. Therefore, and given the brevity of his visit, he was unable to determine whether mental decline was a new development or was pre-existing, and did not assess whether CI, whenever found, was new. This information was obtained later from the RMA data and added to the 4AT score for patients diagnosed with CI, i.e., the information whether the CI was new or not is equal in RMA and 4AT (with the exception of false negative and false positive RMAs). Specifically, after 4ATs were performed, the principal investigator checked the RMA record, and added to the 4AT whether a CI was acute or chronic, according to the staff’s findings. In cases where the 4AT was > 0 but RADAR or MOYB were negative, the principal investigator inquired about the matter with the family members, and completed the 4AT accordingly. Delirium cases were scored as false negative RMA. If the family did not notice the slight CI found in the 4AT, the impairment was considered as chronic.

### Statistical analysis

Results were presented in binary form - positive or negative for delirium. In addition to new onset or fluctuating course, both positive RADAR and MOYB were required for a positive RMA test. For 4AT, the “gold-standard” in this study, a score of at least 1 in addition to the 4 points scored for the acute event (i.e. a total of 5 points or more) was required for the diagnosis of delirium. From a clinical point of view, a high sensitivity (> 90%), and therefore a low false negative rate (low β error) was considered to be most important to avoid overlooking patients with delirium. The sample size required for a sensitivity of 95% and a 95% confidence interval was calculated according to Buderer et al. [26], and was *n* = 365, assuming delirium prevalence of > 20% in elderly patients hospitalized in internal medicine wards [3]. In addition to RMA sensitivity, specificity, and positive and negative predictive values, the Pearson correlation, Altman-Bland plot, non-inferiority test and ROC analysis were used to compare RMA and 4AT.

## Results

Three hundred and eighty-four patients were included in the study. Their age, gender and test results are shown in Table 2. In total, 98 patients (25.5%) were found to suffer from delirium, based on a positive 4AT. The 286 subjects in whom 4AT was negative (4AT-) included 127 patients with a 4AT score > 0 in whom cognitive impairment was identified as pre-existing (chronic cognitive impairment [CCI], probably dementia). The male-female distribution was similar in both groups. Patients with 4AT + were significantly older than those with 4AT- (*p* < 0.001). Per definition, the average score of patients with 4AT- was far lower than that of 4AT+. The positive scores in patients with 4AT- were due to the scores of patients with CCI, including those with rather mild impairment, i.e. 1 point.

**Table 2:** Patient gender, age and test results

	4AT + (delirium)	4AT -
n (% of all)	98 (25.5%)	286 (74.5%)
Female (%)	63.3%	64.6%
Age (years, mean ± SD)	85.2 ± 6.8	81.8 ± 7.2*
4AT Score (mean ± SD)	7.56 ± 1.55	1.26 ± 1.89
RMA+ (% 4AT)	92 (93.9% true positive)	5 (1.7% false positive)
CCI	0	127

CCI– chronic cognitive impairment. * - *p* < 0.001

The RMA test was positive in 92 of the 98 patients with positive 4AT, but also in 5 patients with negative 4AT. Four cases of delirium were overlooked by the nurses, and another 2 by the ward residents. The false positive RMA tests were, by definition, due to both. Nurses reported a positive RADAR in 22 patients without delirium or CCI. In most patients with a false positive RADAR the ward resident ruled out delirium based on a normal MOYB test.

The results of the 4AT correlated highly (0.924) with those of the RMA test, and were identical in 97% of patients. Since the result in both tests was either positive or negative, we used a paired binary test (McNemar) to test the hypothesis that both tests are identical. The result (*p* = 0.99) shows that the tests are indeed almost identical.

Table 3 shows the sensitivity, specificity, and positive and negative predictive values of RMA relative to 4AT. RMA had a sensitivity of 93.9% (CI 89.1, 98.6), and specificity of 98.3 (CI 96.7, 99.8), with the other parameters reaching 95% and higher. In accordance with these results, ROC analysis indicated an excellent match, with an AUC of 0.964 (CI 0.937, 0.991).

**Table 3:** Sensitivity, specificity and predictive values for RMA and its components. TP– true positive; FP– false positive; TN– true negative; FN– false negative; CI– confidence interval; Sens.- Sensitivity; Spec.- Specificity; NPV– negative predictive value; PPV– positive predictive value. *N* = 384; 4AT positive = 98; 4AT negative = 286

	TP (n)	FP (n)	TN (n)	FN (n)	Sens. % (95% CI)	Spec. % (95% CI)	NPV % (95% CI)	PPV % (95% CI)
RADAR	94	104	182	4	95.8 92.0, 99.8))	63.6 58.1, 69.2))	97.8 95.8, 99.9))	47.5 (40.5, 54.4)
RADAR + MOYB	92	80	206	6	93.9 (89.1, 98.6)	72.0 (66.8, 77.2)	97.2 (94.9, 99.4)	53.5 (46.0, 60.9)
**RMA**	92	5	281	6	**93.9** (89.1, 98.6)	**98.3** (96.7, 99.8)	**97.9** (96.3, 99.6)	**94.8** (90.4, 99.2)

**Fig. 1:**
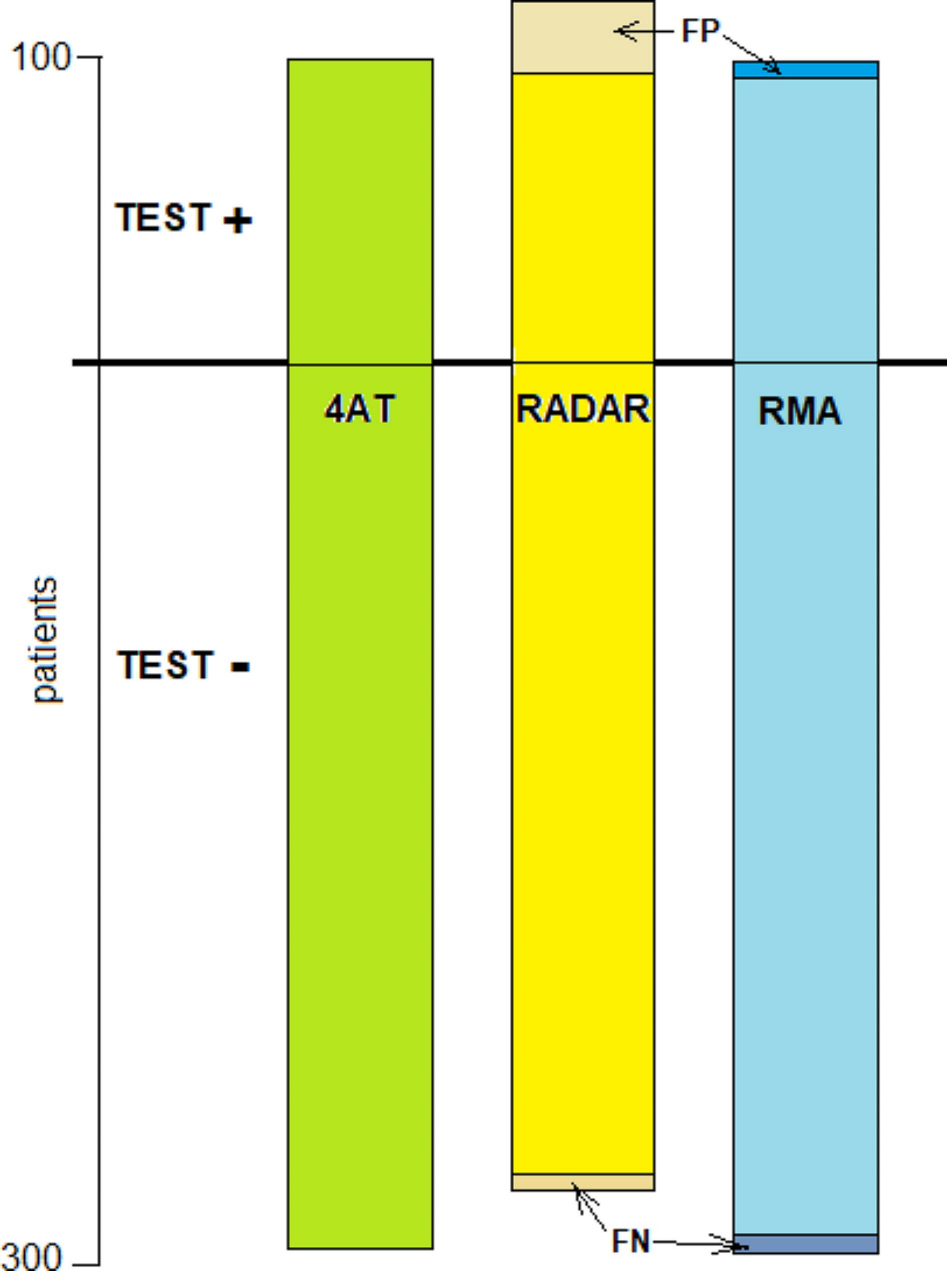
Comparison of the RADAR and RMA tests to 4AT.FP and FN– false positive and false negative, respectively. The FN of RADAR refers to missed patients with cognitive impairment, while that of RMA indicates missed delirium

Figure 1 compares the results of RMA (assessing delirium) and RADAR (assessing CI), to 4AT. It can be seen that compared to 4AT, RADAR had a very high sensitivity (the low false negative value refers to missed patients with CI). RMA had only marginal false positive and false negative results, reduced the number of false positive finding in RADAR, and focused the results to delirium,.

RADAR indicates only the presence of CI, as it does not include the components of recency of the impairment and the presence of an attention/concentration deficit required for the diagnosis of delirium. Therfore, its high sensitivity (95.8%) was associated with a rather low specificity for delirium (63.6%). Adding MOYB still assesses only CI. RADAR + MOYB had a sensitivity of 93.9% (due to 2 false negative of the physicians), but improved the specificity for delirium to 72.0% by correcting most of the nurses’ false positive results. Only after adding the 3rd component, whether the CI was new, the specificity of RMA increased to 98.3% (Table 3).

For the purpose of a more detailed comparison between the evaluations of the department staff (RMA) and the rater (4AT), we used the parameters that were identical in both tests, i.e., alertness (scored in R&M by the nurses), and MOYB (scored in R&M by the residents). The correlation between alertness in 4AT and RADAR was 0.48. Although highly significant (*p* < 0.00001), the result indicates that the assessment of alertness may be quite variable. Concordance between the nurses’ and rater’s assessment of alertness was 86.9%. Concordance between the department residents’ and rater’s assessment of MOYB (for patients in whom MOYB was administered) was 87.2%.

Non-inferiority studies are probably the most widely used method of comparing a new diagnostic test with an older, widely accepted one. Using a paired, binary, one-tailed test, the mean difference between the 4AT and RMA was 0.0026 and the confidence interval was 0.012, 0.0168, far under the 0.05 limit, thus confirming the non-inferiority of RMA compared to 4AT.

Another extensively used method to compare measurement methods in medicine (i.e. a new test vs. a gold-standard) is the Altman-Bland plot, which is a mean-difference chart. In the present study, apart from a slight deviation in two outliers, the other 382 patients were within the 95% range (± 1.96SD) of the mean differences between the test results. This finding indicates a good match, and together with the high sensitivity and specificity of RMA, it indicates that the methods can be used interchangeably.

## Discussion

This study shows that there is a striking similarity between the results of the RMA and 4AT, so much so that in practice both tests can be used interchangeably. Clinically, the most important result was the sensitivity of the RMA, which reached 94% along with 98% specificity compared to the 4AT, indicating that only a few patients with delirium may have been overlooked by the department staff.

The high correlation between the tests is not surprising. Although the CAM may be more widely used, the 4AT has undergone similar validation, and was a suitable comparator for the RMA, as the tools used in the RMA and 4AT are almost identical. Assessment of alertness was required in both tests. Although in the 4AT it was evaluated by a rater-physician and in the RMA it was evaluated by nurses, a previous comparison showed that both rate delirium similarly [20]. MOYB was used in both tests, and when the 4AT rater identified a cognitive impairment, he was informed by the principal investigator whether it was new or chronic. Accordingly, our comparison between the 4AT and RMA can be considered almost as an assessment of inter-rater variability. The main difference between the tests was that, as in the ultra-brief screening [17–20], the AMT4 was not included in the RMA. Inadequate attention/concentration detected on the MOYB was considered sufficient for the department residents to confirm the nurses’ impression of cognitive impairment. In contrast to the 4AT, the RMA was performed every day by staff who had previously been in contact with most patients, making it easier for them to perceive a change in the patent’s cognitive state, even without the AMT4. Although in this study we took the 4AT as the gold standard and our goal was to validate the non-inferiority of RMA compared to 4AT, 4AT has its own limitations, and has been found to have a pooled sensitivity and specificity of 0.88 [27]. Therefore, it is reasonable to assume that the department staff’s assessments were occasionally more accurate than those of the rater, who met the patients for the first time when administering the 4AT. It should be noted that we did not differentiate between delirium alone and delirium superimposed on dementia due to the difficulty in distinguishing between these conditions during a short hospital stay [28, 29].

This study was not designed to assess whether the RMA may be a better diagnostic tool than the 4AT. Although the tests are equivalent, the RMA has at least two important advantages. First, although the benefit of early detection of emergent delirium is widely known and accepted, relatively few internal medicine and surgery wards appear to perform routine daily assessments [2, 6]. This is not due to the complexity of delirium detection, as short, effective assessment tools can be administered in a few minutes [13–20]. Nevertheless, doctors tend to avoid these tests due to time constraints, and dedicated personnel with adequate skills and expertise are expensive. The RMA, however, administered as part of routine ward practice, appears to be more user-friendly and is more likely to be correctly administered, as the nurse needs only a few seconds to mark her impression of the patient’s mental state in the electronic record [22, 23]. An MOYB takes less than a minute [13, 18–20], and only needs to be administered in patients with a positive RADAR. Secondly, but equally importantly, the R&M helps raise awareness of delirium when it is administered by ward staff. A clinician who has been warned of delirium and has recognized the initial signs during the morning round can implement the necessary investigations and treatment without delay.

This study has several limitations. First, and most importantly, the study was designed to compare the RMA with the 4AT. Therefore, only completed RMA tests performed within the defined time frame were used for the purpose of comparison. We did not test staff compliance with RMA testing, a factor that is obviously critical to identify the development of delirium. On the other hand, using only patients with all required data is not expected to have introduced a bias that could affect the comparison of the tests: RMA was performed daily, and the 4AT rater did not know the patients. Additional follow-up is required to identify other actions that may improve compliance with the relevant clinical guidelines [29–32].

The RMA has another potential shortcoming, insofar as a false negative RADAR assessment by the nurse means that the ward physician is not required to administer the MOYB. Therefore, the sensitivity of RMA was limited by RADAR.We accepted this drawback in order to simplify the RMA. To compensate, the nurses were instructed to increase the sensitivity of their assessment, even at the expense of specificity. This approach resulted in 22% of false positive RADARs. However, after adding the MOYB, the final RMA specificity and negative predictive values remained nevertheless very high, around 98%. Another concern could be the exclusion of nursing home patients with advanced dementia. However, comparing binary tests in such patients is not useful, since they will be found positive in any test. Also, although RMA and 4AT were performed close to each other, it cannot be ruled out that short fluctuations in the state of consciousness could have contributed to differences between the test results. The fact that the information about the recentness of CI, whenever found, was the same in 4AT and RMA, may be considered as problematic, as this crucial component of the test of interest (RMA) was the same as of the reference test (4AT). However, we consider the information regarding the acuteness of CI as not related to the CI itself, but rather to the sources of the information, and therefore should not be part of the assessment of the validity of RMA. Finally, 4AT’s sensitivity and specificity are both 88% [27], which may bias the true R&M’s sensitivity and specificity estimates. However, as noted above, it is reasonable to assume that the department staff’s assessments were occasionally more accurate than those of the rater, and together with occasional short fluctuations in consciousness, we believe that RMA and 4AT estimate delirium equally. In addition, considering the high sensitivity and specificity of RMA when compared to 4AT, even if we assume that in all patients 4AT was always correct, still the “true” sensitivity and specificity of RMA will be very good, reaching 0.83 and 0.85, respectively.

## Conclusions

The RMA test is based on the basic concept of assessing the presence of delirium by the department staff during routine work. We found that nurses are most effective in screening patients for possible delirium. The pre-screening reduced the number of elderly patient that had to be further evaluated by the clinicians, and enabled to use a simple and short test to reach a reliable diagnosis. Our results indicate that RMA is equivalent and non-inferior to 4AT. In addition, the collaboration between nurses and physicians may improve implementation of RMA and enhance awareness of delirium.

## Data Availability

The datasets used and/or analysed during the current study are available from the corresponding author on reasonable request and with permission of Bnai Zion Medical Center.

## Declarations

### Trial registration

NCT05836714, Apr. 28, 2013 (retrospectively registered).

## Abbreviations

RMA: RADAR&MOYB
RADAR: Recognizing Acute Delirium As part of your Routine
MOYB: Months Of the Year Backwards
4AT: Attention, Abbreviated mental test, Alertness, Acute onset test
CI: cognitive impairment
CCI: chronic cognitive impairment
